# Unmasking the mimic: vertebral alveolar echinococcosis diagnosed by metagenomic next-generation sequencing

**DOI:** 10.1007/s15010-025-02717-3

**Published:** 2025-12-21

**Authors:** Tassilo Kruis, Marion Wassermann, Barbara Graf, Katharina Lührig, Peter Menzel, Rolf Schwarzer, Johannes Ziegler, Caroline Isner

**Affiliations:** 1 Labor Berlin Charité Vivantes GmbH, Berlin, Germany; 2 Department of Infectious Diseases, Vivantes Auguste-Viktoria-Klinikum, Berlin, Germany; 3 Parasitology Unit, Institute of Biology, University of Hohenheim, Stuttgart, Germany; 4 Department of Infectious Disease, Respiratory Medicine and Critical Care, Charité-Universitätsmedizin Berlin, Berlin, Germany

**Keywords:** Echinococcus multilocularis, Vertebral alveolar echinococcosis, Spinal tuberculosis, Metagenomic next-generation sequencing

## Abstract

A Siberian woman in her forties presented to a public hospital in northeastern Germany with chronic back pain and a paravertebral mass, initially misdiagnosed as spinal tuberculosis. Repeated biopsies and metagenomic next-generation sequencing (mNGS) ultimately confirmed vertebral alveolar echinococcosis. Haplotype analysis revealed a novel Asian-cluster variant, supporting the presumed origin of infection.

## Introduction

Alveolar echinococcosis (AE), caused by the tapeworm *Echinococcus multilocularis*, is a rare but potentially fatal zoonotic disease affecting the northern hemisphere. Current epidemiological data demonstrate a doubling of AE incidence rates in Europe from 0.063 (1997–2023) to 0.121 per 100000 (2021–2023), with high-risk clusters in the alpine region (Switzerland, eastern France, southern Germany, and western Austria) and the baltic region (Latvia, Lithuania, and Poland) [1, 2]. Regions of high endemicity are also present in parts of the Russian federation and north-eastern Asian countries, which is relevant in context of migration from these areas to central Europe [1, 3].

The liver is the primary site of AE infection in over 95% of cases, whereas extrahepatic involvement is rare. Vertebral AE (VAE), a rare osseous manifestation, accounts for only 0.02–1.0% of all AE cases [4].

Imaging techniques such as computer tomography (CT) and magnetic resonance imaging (MRI) are essential for the diagnosis and management of AE but are often nonspecific, particularly in extrahepatic lesions. Findings frequently mimic those of other diseases such as spinal tuberculosis, tumors, or bacterial and fungal abscesses [4, 5]. Thus, final diagnosis relies on serology using enzyme-linked immunosorbent assays (ELISA) against Em2/Em18 antigens, histopathology, and polymerase chain reactions (PCR) on tissue samples [4].

Metagenomic next-generation sequencing (mNGS) has recently proven effective for AE diagnosis using tissue or blood samples [6–9]. However, AE’s rarity complicates diagnosis, particularly in atypical cases like VAE. Spinal tuberculosis is an important differential diagnosis, and misdiagnosis can result in severe, even fatal outcomes [5, 7, 10].

This report presents a VAE case initially misdiagnosed as spinal tuberculosis. The correct diagnosis was established through repeated invasive sampling, histopathology, and mNGS, highlighting both the diagnostic challenges of VAE and the high utility of mNGS in identifying rare infections. Beyond pathogen identification, genetic data can also contribute to tracing the likely origin of infection through comparative haplotype analysis [9, 19]. In the present case, such analysis was performed and supported the presumed geographic origin.

## Case presentation

A woman in her forties with no significant medical history presented to the orthopedic outpatient clinic with chronic lower back pain and intermittent sharp pain radiating to the right leg, persisting for 8 months. Aside from this and a tapping pain over the lumbar spine and sacroiliac joint, no other abnormal findings were noted. MRI revealed a large paravertebral cystic mass in the thoracolumbar region with partial destruction of the lateral vertebral bodies. She denied systemic symptoms, including weight loss, fever, night sweats, respiratory complaints, or urinary or fecal incontinence.

The patient was born and raised in a small village in Novosibirsk Oblast, Siberia, Russia, in a household with a subsistence garden, where wild forest fruits were also consumed. Additional exposure risks, such as hunting activities or dog ownership, were not known. She had worked at a military base before the onset of the war in Ukraine and her relocation to a metropolitan area in northeastern Germany. She reported no travel outside Siberia or Germany in the past ten years. Her medical, surgical, and social history was otherwise unremarkable: she denied substance use, prior injections or surgeries, and any family history of cancer or tuberculosis.

**Fig. 1:**
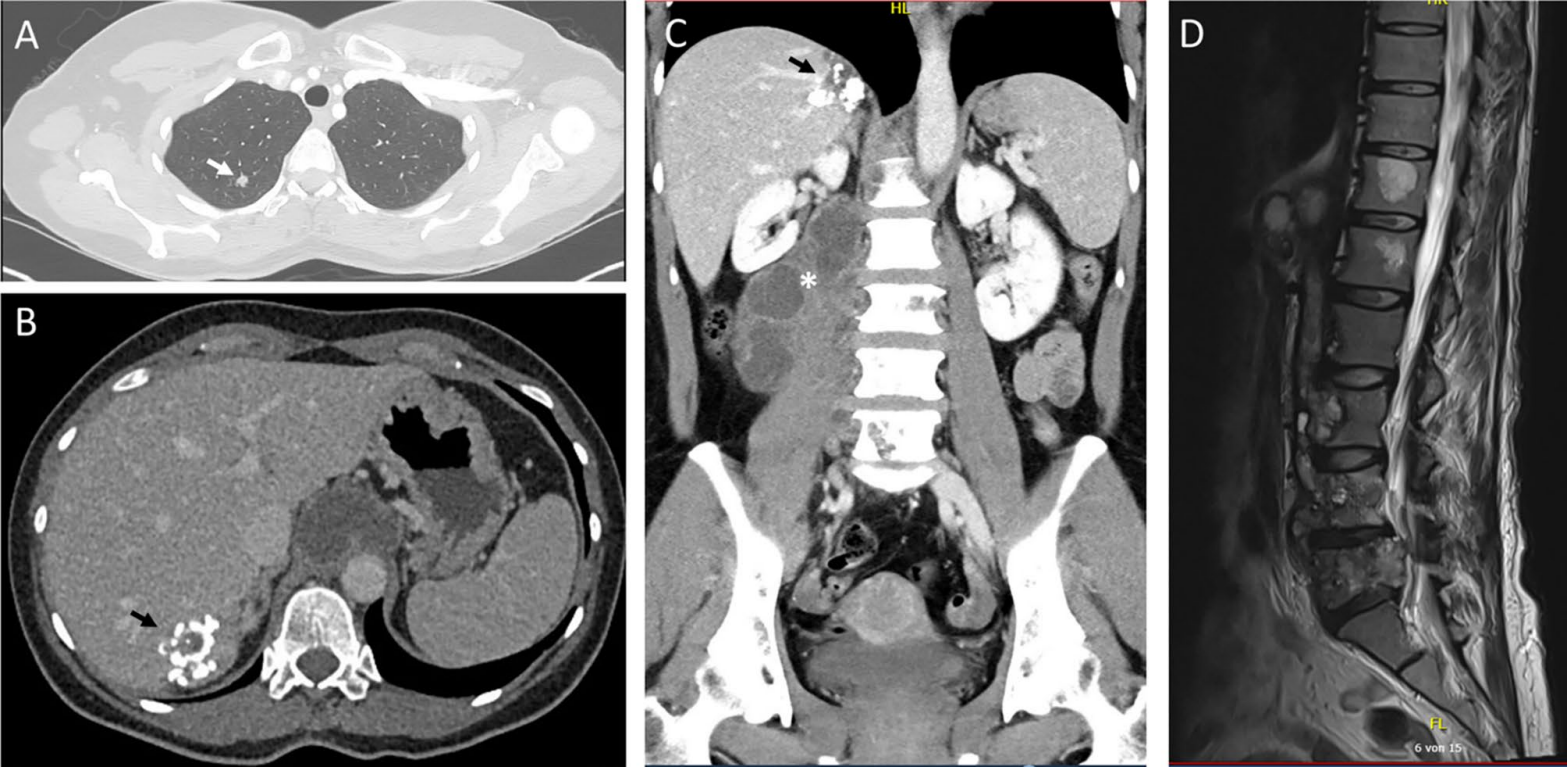
**A**, **B** Axial CT images at initial presentation show dense pulmonary nodules (white arrow) and a ring-shaped hepatic calcification (black arrow). **C** Coronal CT reconstruction depicts the hepatic lesion and an approximately 11 × 4 cm hypodense, multilobulated mass extending from the interaortocaval region along the psoas muscle to the aortic hiatus (asterisk), with osteolysis of lumbar vertebrae 3–5. **D** MRI after eight weeks of anti-tuberculosis therapy reveals progressive osteodestruction with a small polycystic or honeycomb appearance and inflammation involving the ventral epidural space

Initial laboratory tests showed normal blood counts (white blood cells 5.9 × 10^9^/L, eosinophils 0.03 × 10^9^/L), but mildly elevated aspartate aminotransferase (40 U/L), lactate dehydrogenase (327 U/L), and C-reactive protein (24.5 mg/L). Total bilirubin (0.4 mg/dL), alanine aminotransferase (26 U/L), alkaline phosphatase (96 U/L), and gamma-glutamyl transferase (35 U/L) were within normal limits. HIV serology was negative. CT revealed a hypodense, polylobulated mass extending from the interaortocaval area along the psoas muscle to the aortic hiatus, multiple small dense nodules in both lungs, and a ring-shaped hepatic calcification (Fig. 1).

Radiological findings initially suggested tuberculous spondylitis, prompting CT-guided drainage of a presumed psoas abscess and puncture of the fifth lumbar vertebra. Purulent material was submitted for histopathological and microbiological analysis, including extended aerobic and anaerobic cultures (14 days), fungal cultures, *Mycobacterium tuberculosis* (*Mtb*)-PCR (Cobas MTB assay, roche diagnostics), and mycobacterial cultures. Histopathology revealed granulocytic necrosis with multinucleated macrophages but no evidence of malignancy, fungal infection, or mycobacteria; both auramine staining and *Mtb*-PCR were negative. Nonetheless, a standard anti-tuberculosis regimen (rifampin 600 mg q24 h, isoniazid 300 mg q24 h, ethambutol 400 mg q24 h, pyrazinamide 500 mg q24 h) was initiated, with empiric levofloxacin (500 mg q12 h) added to cover common bacterial etiologies of spinal abscesses. Over the next 8 weeks, the patient’s condition deteriorated, with worsening right leg pain while walking. Follow-up MRI showed progressive lumbar osteodestruction and a slight increase in the multilocular abscess size. Antimicrobial therapy was stopped when mycobacterial cultures remained negative.

A subsequent CT-guided drainage yielded 400 mL of purulent fluid, which was microbiologically negative. Empiric antimicrobial therapy with ceftriaxone (2 g q24 h) and metronidazole (400 mg q8 h) for 2 weeks, along with doxycycline (100 mg q12 h) for 6 weeks, was administered; however, no clinical improvement was observed, and the therapy was subsequently discontinued. Follow-up CT showed no change, prompting further diagnostic and therapeutic aspiration of the abscess.

**Fig. 2:**
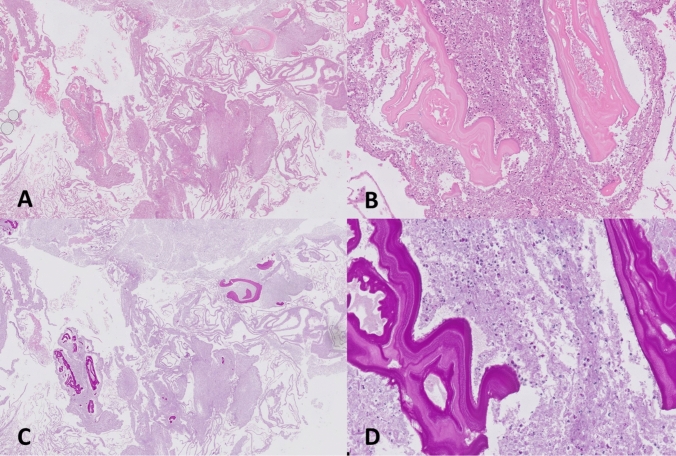
Hematoxylin and eosin (HE) stains (**A**, **B**) and periodic acid–Schiff (PAS) stains (**C**, **D**) of abscess tissue in which *E*. *multilocularis* DNA was detected by mNGS. Sections show *Echinococcus* cyst walls at different magnifications (40× in **A**, **C**; 200× in **B**, **D**) with the characteristic concentric layering. Histopathological specimens courtesy of Sandra Richter, department of pathology, Vivantes hospital am Urban, Berlin

Two newly obtained abscess samples were analyzed by mNGS. Whole-genome sequencing identified 1413 and 3419 reads (out of 11.2 million and 30.8 million total reads) classified as *Echinococcus multilocularis*, which was confirmed by histopathological evidence of a metacestode (Fig. 2) and positive serology including ELISA targeting Em2/Em18 antigens (result 4.7, cutoff range 0.9–1.1; *E*. *multilocularis* ELISA, bordier affinity products SA).

**Fig. 3:**
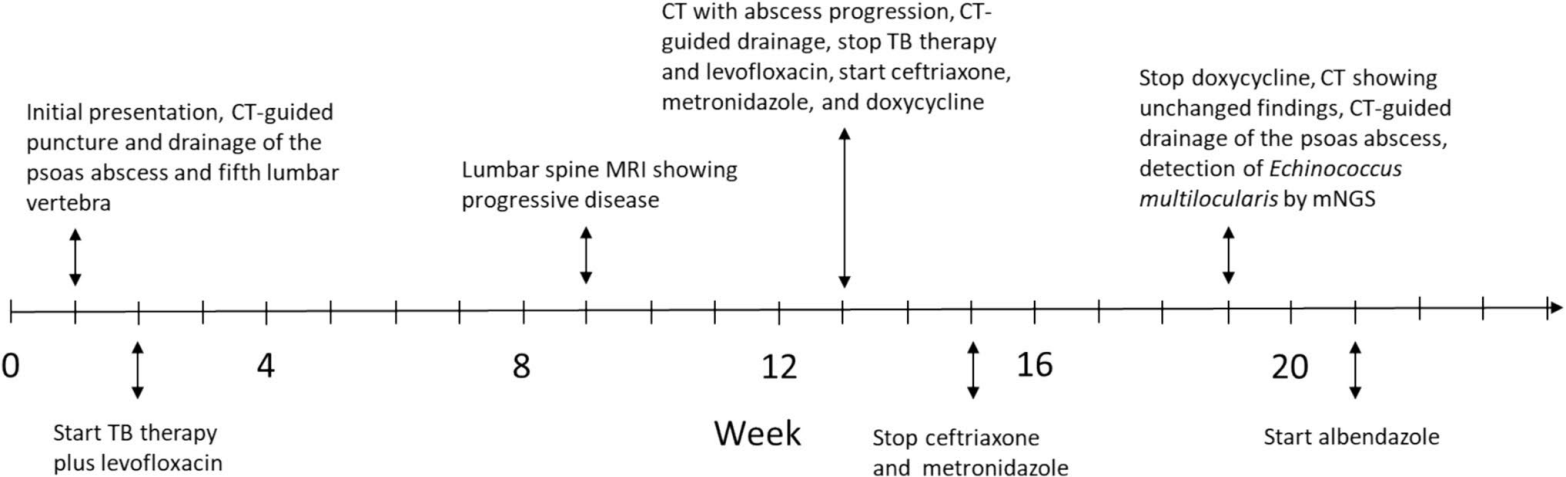
Key events in the disease course up to diagnosis of vertebral alveolar echinococcosis. *CT* computer tomography, *MRI* magnetic resonance imaging, *TB* tuberculosis, *mNGS* metagenomic next generation sequencing

Albendazole therapy (400 mg q12 h) was initiated, and the patient was referred to an echinococcosis center in southern Germany. Surgical removal of the metacestode was considered too high-risk due to potential complications; thus, a watchful waiting strategy was adopted. During albendazole therapy, a slight increase in alanine aminotransferase (83 U/L) was noted, while total bilirubin (0.3 mg/dL) remained within the normal range. After 6 months of therapy, the patient reported increasing fatigue, sleep disturbances, and diffuse hair loss, while imaging showed marked regression of the psoas abscess, stable osteodestruction, and decreasing inflammatory changes, so albendazole therapy was continued at 200 mg q12 h. Figure 3 illustrates the temporal course of the disease up to the diagnosis of VAE.

## Positioning of specimen in haplotype network

**Fig. 4:**
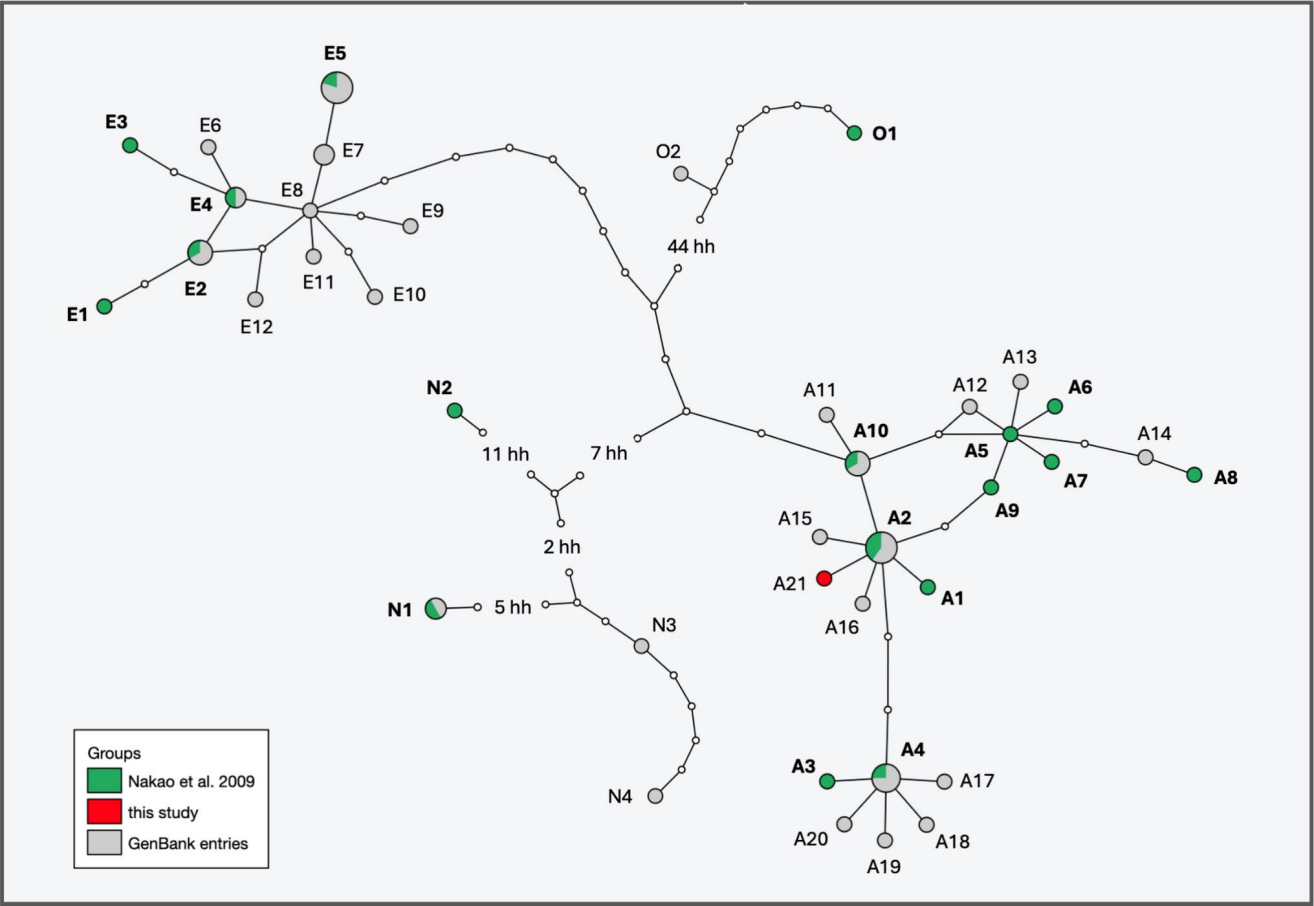
Haplotype network of *E*. *multilocularis* based on concatenated sequences of *cob*, *nad2*, and *cox1* genes (3588 bp). Haplotypes originally defined by Nakao et al. [7] are shown in green and bold; additional genetic variants were included, limited to one identical haplotype per country or region of origin. The haplotype identified in the patient is located within the Asian cluster and highlighted in red. Circle sizes reflect haplotype frequencies, while small colorless circles represent hypothetical haplotypes (hh)

Comparison with GenBank entries revealed that the *Echinococcus multilocularis* variant detected in the patient has not been previously reported. Haplotype network analysis based on the mitochondrial cytochrome b (*cob*), NADH dehydrogenase subunit 2 (*nad2*), and cytochrome c oxidase subunit 1 (*cox1*) genes (3558 bp) placed this variant within the Asian cluster, with haplotype A2 as its closest relative, differing by a single nucleotide substitution (Fig. 4). Haplotype A2 has previously been reported in Russia (Altai and Ryazan Oblasts), Kazakhstan, China (Xinjiang), and Alaska (St. Lawrence Island).

Geographically, the Altai region, Kazakhstan, and Xinjiang are located near the presumed origin of infection in Novosibirsk Oblast. Additionally, haplotypes A1, A10, and A15, which differ by two nucleotide substitutions, have also been reported in the Altai region and Kazakhstan (Table 1). This phylogeographic evidence strongly suggests that the infection was acquired most likely at the patient’s former place of residence.

**Table 1:** Haplotypes, geographic origin, GenBank accession numbers, and references used for haplotype network analyses (E: European, N: North American, O: Mongolian, A: Asian)

Haplotype	Geographic origin	GenBank accession numbers			References
		*cob*	*nad2*	*cox1*	
E1	Austria	AB461395	AB461403	AB461412	[11]
E2	Germany	AB461395	AB461403	AB461414	[11]
	France	OQ599957	OQ599957	OQ599957	[19]
	Luxembourg	OQ599958	OQ599958	OQ599958	[19]
E3	France	AB461396	AB461404	AB461413	[11]
E4	Belgium	AB461395	AB461404	AB461414	[11]
	France	OQ599960	OQ599960	OQ599960	[19]
E5	Slovakia	AB461397	AB461405	AB461414	[11]
	Switzerland	OR911405	OR911405	OR911405	[20]
	Sweden	OR911423	OR911423	OR911423	[20]
	Poland	OR911438	OR911438	OR911438	[20]
	France	OQ599966	OQ599966	OQ599966	[19]
E6	France	OQ599941	OQ599941	OQ599941	[19]
E7	Switzerland	OR911422	OR911422	OR911422	[20]
	France	OQ599963	OQ599963	OQ599963	[19]
E8	Poland	OR911433	OR911433	OR911433	[20]
E9	France	OR911393	OR911393	OR911393	[20]
E10	Canada (British Columbia)	OR911407	OR911407	OR911407	[20]
E11	Switzerland	OR911416	OR911416	OR911416	[20]
E12	France	OR911387	OR911387	OR911387	[20]
N1	Alaska (St. Lawrence Island)	AB461400	AB461409	AB461418	[11]
N2	USA (Indiana, South Dakota)	AB461401	AB461410	AB461419	[11]
N3	Norway (Svalbard)	OR911421	OR911421	OR911421	[20]
N4	Russia (Yakutia)	OR911451	OR911451	OR911451	[20]
O1	China (Inner Mongolia)	AB461402	AB461411	AB461420	[11]
O2	Russia (Irkutsk Oblast)	OR911453	OR911453	OR911453	[20]
A1	Kazakhstan	AB461398	AB461406	AB461415	[11]
A2	Kazakhstan	AB461398	AB461406	AB461416	[11]
	Alaska (St. Lawrence Island)	AB461398	AB461406	AB461416	[11]
	Russia (Altai)	OR911443	OR911443	OR911443	[20]
	Russia (Ryazan Oblast)	OR911448	OR911448	OR911448	[20]
	China (Xinjiang)	OP628494	OP628494	OP628494	Guo, unpublished
A3	Japan	AB461399	AB461407	AB461416	[11]
A4	Japan	AB461398	AB461407	AB461416	[11]
	Alaska (St. Lawrence Island)	AB461398	AB461407	AB461416	[11]
	Canada	OR911406	OR911406	OR911406	[20]
	Japan	LC720785	LC720785	LC720785	[21]
	Alaska (St. Lawrence Island)	LC720790	LC720790	LC720790	[21]
A5	China (Sichuan)	AB461398	AB461408	AB461417	[11]
A6	China (Sichuan)	AB461398	AB461408	AB477011	[11]
A7	China (Sichuan)	AB461398	AB461408	AB477012	[11]
A8	China (Sichuan)	AB477009	AB461408	AB477010	[11]
A9	China (Sichuan)	AB461398	AB461408	AB461416	[11]
A10	China (Sichuan)	AB461398	AB461406	AB461417	[11]
	Poland	OR911432	OR911432	OR911432	[20]
	Russia (Altai)	OR911440	OR911440	OR911440	[20]
A11	Armenia	OR911444	OR911444	OR911444	[20]
A12	China (Qinghai)	OR911426	OR911426	OR911426	[20]
A13	Japan	LC720770	LC720770	LC720770	[21]
A14	France	OQ599968	OQ599968	OQ599968	[19]
A15	Russia (Altai)	OR911441	OR911441	OR911441	[20]
A16	Russia (Ryazan Oblast)	OR911450	OR911450	OR911450	[20]
A17	Japan	LC720780	LC720780	LC720780	[21]
A18	Japan	LC720778	LC720778	LC720778	[21]
A19	Japan	LC720772	LC720772	LC720772	[21]
A20	Japan	LC720763	LC720763	LC720763	[21]
A21	Russia (Novosibirsk Oblast)	PV404220	PV404219	PV356069	This study

## Methods

### Metagenomic next generation sequencing

Genomic DNA was extracted from patient samples, eluted in 50 μL elution buffer using the DNeasy PowerSoil Pro-Kit (Qiagen), and quantified using Quant-iT PicoGreen dsDNA quantification kit (thermo scientific). Sequencing-ready shotgun DNA libraries were prepared with the QiaSeq FX (Qiagen) kit according to the manufacturer’s protocol (https://www.qiagen.com/kw/resources/download.aspx?id=9ad037fd-3fc4-487a-a13f-8d14776af956&lang=en). Briefly, genomic DNA was enzymatically fragmented, and sequencing adapters containing sample-specific barcode sequences were ligated to both ends of the DNA fragments. Libraries were sequenced on a NextSeq 1000 with 2 × 250 bp paired-end reads. After adapter trimming, reads were classified with kraken using its standard database (date 1/12/2024) to remove human sequences, followed by classification of the remaining non-human reads using a custom database comprising several eukaryotic pathogens, including the three *Echinococcus* species *E*. *multilocularis* (NCBI accession GCA_000469725.3, *E*. *oligarthrus* (GCA_900683695.1), and *E*. *granulosus* (GCF_000524195.1).

### Conventional PCR and haplotype analysis

Due to incomplete gene fragments from shotgun sequencing, conventional PCR and Sanger sequencing were performed to obtain full-length sequences of *cob*, *nad2*, and *cox1* genes, as previously described [11], to determine the haplotype. A haplotype network was constructed to assess whether genetic data supported the presumed origin of infection. For this purpose, *Echinococcus multilocularis* sequences representing the European, Asian, North American, and Mongolian clades were retrieved from GenBank (Table 1). The network was generated using TCS 1.2 [12] based on statistical parsimony [13] and visualized with tcsBU [14].

## Discussion

This case of a woman with a thoracolumbar paravertebral cystic mass, initially misdiagnosed as spinal tuberculosis, illustrates the diagnostic challenges in distinguishing VAE from more common spinal pathologies. Despite the availability of noninvasive diagnostic options such as Em2/Em18 antigen ELISA, the rarity of VAE often leads to delayed diagnosis [7, 9, 10]. Both VAE and spinal tuberculosis show an insidious clinical course with overlapping CT/MRI findings, including osteolytic lesions and bone destruction, paravertebral soft tissue masses, multi-segment spinal involvement, and T1-hypointense/T2-hyperintense MRI signals [4, 5, 15]. Siberia is regarded as a high-endemicity area for AE; however, precise epidemiological data on incidence rates remain limited [1, 3]. Nevertheless, the incidence of tuberculosis (48 per 100,000) is likely substantially higher than that of AE [16], reinforcing a diagnostic bias towards the more prevalent condition.

The present case demonstrates similarities with recent reports from China [7–9], where AE has one of the world’s highest prevalence rates [1, 3]. In two of three cases, patients were initially misdiagnosed with tuberculosis and received empirical anti-tuberculosis therapy prior to definitive diagnosis. Histopathological examination alone proved insufficient for confirming AE, likely due to limited specimen size or quality in extrahepatic echinococcosis and the presence of non-specific findings such as granulomatous inflammation with necrosis, which lack pathognomonic features.

Furthermore, serological cross-reactivity between *E*. *multilocularis* and other cestodes, such as *Taenia solium* endemic in the same regions, significantly reduces diagnostic specificity. This constraint, combined with the inability to distinguish active infection from residual seropositivity due to past exposure, substantially compromises the reliability of conventional serological testing [7–9]. Another limitation is that immune status can influence serological detection, potentially leading to false-negative results in immunosuppressed AE patients [17].

Although VAE and spinal tuberculosis share similar imaging findings, several features may aid differentiation. Spinal tuberculosis typically demonstrates a paradiscal pattern with disc preservation until late stages, subligamentous spread across multiple vertebral levels, and characteristic smooth-walled “cold abscesses” with paravertebral calcifications, predominantly at the thoracolumbar junction [15]. In contrast, alveolar echinococcosis may display a distinctive multicystic “honeycomb” or “grape bunch-like” morphology, and clustered microcalcifications with slight rim enhancement but minimal internal enhancement (Table 2). This characteristic appearance results from metacestodes secreting proteolytic enzymes that facilitate tissue breakdown and invasion, combined with a unique infiltrative growth pattern characterized by exogenous budding and daughter vesicles formation [4, 5]. Despite these differences, definitive diagnosis requires the integration of imaging with epidemiological history, serological testing, and histopathological confirmation, as radiology alone cannot reliably distinguish these entities.

**Table 2:** Comparison of key imaging features of vertebral alveolar echinococcosis and spinal tuberculosis based on [4, 5, 15, 22]

Imaging feature	Vertebral alveolar echinococcosis	Spinal tuberculosis
Systemic distribution	Primary hepatic with extrahepatic metastases (lung, brain, bone)	Primary pulmonary/nodal; contiguous spread along spine
Typical spinal levels	Lower thoracic, upper lumbar	Lower thoracic, thoracolumbar junction, lumbar
Overall lesion pattern	Irregular, infiltrative, tumor like mass; multicystic/honeycomb	Destructive lesions with soft tissue mass/abscess
Internal architecture	Multivesicular “honeycomb”, many small cysts/vesicles	Caseous necrosis, liquefied centers in abscesses
Enhancement pattern	Little/no internal enhancement; mild peripheral fibrotic rim	Rim enhancing abscesses; enhancing granulomatous tissue
Calcifications	Very common; scattered, plaque like or ring like calcifications	Possible but less specific; usually in chronic/treated disease
Bone destruction	Irregular, heterogeneous osteolysis, expansive destruction	Fragmentary/lytic vertebral body destruction
Paravertebral component	Infiltrative, multicystic mass, often calcified	Paravertebral/psoas abscess, well defined rim
Epidural/subligamentous spread	Possible, but less typical and less patterned	Typical long subligamentous spread

The sensitivity of *Mtb*-PCR decreases in paucibacillary infections like spinal tuberculosis, increasing the risk of false-negative results and heightening reliance on clinical judgment. In this case, a calcified hepatic lesion—pathognomonic for primary hepatic involvement in AE—could have provided the critical diagnostic clue. However, this finding was initially overlooked and the diagnosis was only later confirmed through repeated biopsy of the spinal abscess and mNGS.

mNGS is an unbiased diagnostic tool, particularly valuable for identifying non-culturable pathogens or in cases with prior antimicrobial therapy. Although its sensitivity is not superior to culture for common aerobic bacteria, it can be advantageous for anaerobes, *Mtb*, and other difficult-to-culture or unculturable pathogens, including parasites such as *Plasmodium*, *Trypanosoma*, and *Echinococcus* [6, 18].

mNGS has proven effective across various specimen types, including cerebrospinal fluid, aspirates, and tissue samples, offering flexibility in diagnostic approaches based on clinical context and specimen accessibility [7–9]. Detection of parasite-specific circulating cell-free DNA in plasma represents another innovative sequencing-based application for the diagnosis and monitoring of AE. Such liquid biopsies are highly sensitive and specific in AE patients [6].

Theoretically, mNGS can outperform traditional culture methods with turnaround times of 32–36 h [18]. However, achieving such rapid processing requires daily sequencing runs, which are likely cost-effective only in high-throughput facilities. In routine clinical laboratories, samples are usually processed in bi-weekly batches, resulting in turnaround times of up to 7 days.

In summary, this case highlights the importance of considering rare etiologies in spinal abscesses and systematically investigating extrapulmonary findings, even in regions with high tuberculosis prevalence. It demonstrates the particular value of mNGS for rare zoonotic infections such as AE, where conventional methods have significant limitations and diagnostic delays can lead to inappropriate therapy and disease progression.

## Data Availability

Sequencing data are available from the NCBI under BioProject no. [PRJNA1353096] (https:/www.ncbi.nlm.nih.gov/bioproject/PRJNA1353096).
