# Correction: Unmasking the mimic: vertebral alveolar echinococcosis diagnosed by metagenomic next‑generation sequencing

**DOI:** 10.1007/s15010-026-02771-5

**Published:** 2026-04-09

**Authors:** Tassilo Kruis, Marion Wassermann, Barbara Graf, Katharina Lührig, Peter Menzel, Rolf Schwarzer, Johannes Ziegler, Caroline Isner

**Affiliations:** 1 Labor Berlin Charité Vivantes GmbH, Berlin, Germany; 2 Department of Infectious Diseases, Vivantes Auguste-Viktoria-Klinikum, Berlin, Germany; 3 Parasitology Unit, Institute of Biology, University of Hohenheim, Stuttgart, Germany; 4 Department of Infectious Disease, Respiratory Medicine and Critical Care, Charité-Universitätsmedizin Berlin, Berlin, Germany

**Correction: Infection**

10.1007/s15010-025-02717-3

The article “Unmasking the mimic: vertebral alveolar echinococcosis diagnosed by metagenomic next-generation sequencing”, written by Tassilo Kruis^1,2,4^, Marion Wassermann^3^, Barbara Graf^1^, Katharina Lührig^1^, Peter Menzel^1^, Rolf Schwarzer^1^, Johannes Ziegler^2^, Caroline Isner^2^, was originally published under exclusive license to © Springer-Verlag GmbH Germany, part of Springer Nature 2025. As a result of the subsequent decision to publish the article under the open access model, the article’s copyright notice was changed on 13 March 2026 to © The Author(s) 2025, modified publication 2026 and the article is now distributed under a Creative Commons Attribution.

To view a copy of this licence, visit http://creativecommons.org/licenses/by/4.0/.

Open access funding enabled and organized by Projekt DEAL.

**Open Access** This article is licensed under a Creative Commons Attribution-NonCommercial-NoDerivatives 4.0 International License, which permits any non-commercial use, sharing, distribution and reproduction in any medium or format, as long as you give appropriate credit to the original author(s) and the source, provide a link to the Creative Commons licence, and indicate if you modified the licensed material. You do not have permission under this licence to share adapted material derived from this article or parts of it. The images or other third party material in this article are included in the article’s Creative Commons licence, unless indicated otherwise in a credit line to the material. If material is not included in the article’s Creative Commons licence and your intended use is not permitted by statutory regulation or exceeds the permitted use, you will need to obtain permission directly from the copyright holder. To view a copy of this licence, visit http://creativecommons.org/licenses/by/4.0/.

